# How to fix DNA breaks: new insights into the mechanism of non-homologous end joining

**DOI:** 10.1042/BST20220741

**Published:** 2023-10-03

**Authors:** Alex Vogt, Yuan He, Susan P. Lees-Miller

**Affiliations:** 1 Department of Molecular Biosciences, Northwestern University, Evanston, IL, U.S.A.; 2 Interdisciplinary Biological Sciences Program, Northwestern University, Evanston, IL, U.S.A.; 3 Chemistry of Life Processes Institute, Northwestern University, Evanston, IL, U.S.A.; 4 Robert H. Lurie Comprehensive Cancer Center of Northwestern University, Northwestern University, Chicago, U.S.A.; 5 Department of Biochemistry and Molecular Biology, Robson DNA Science Centre and Arnie Charbonneau Cancer Institute, University of Calgary, Calgary, Alberta T2N 4N1, Canada

**Keywords:** DNA double-strand break repair, DNA synthesis and repair, DNA-PK, NHEJ

## Abstract

Non-homologous end joining (NHEJ) is the major pathway for the repair of ionizing radiation-induced DNA double-strand breaks (DSBs) in human cells and is essential for the generation of mature T and B cells in the adaptive immune system via the process of V(D)J recombination. Here, we review how recently determined structures shed light on how NHEJ complexes function at DNA DSBs, emphasizing how multiple structures containing the DNA-dependent protein kinase catalytic subunit (DNA-PKcs) may function in NHEJ. Together, these studies provide an explanation for how NHEJ proteins assemble to detect and protect DSB ends, then proceed, through DNA-PKcs-dependent autophosphorylation, to a ligation-competent complex.

## Introduction

Non-Homologous end-joining (NHEJ) is the major pathway for the repair of ionizing radiation (IR)-induced DNA double-strand breaks (DSBs) in both quiescent and dividing human cells [1]. NHEJ is also required for the processing of immunoglobulin and T cell receptor genes during V(D)J recombination in the adaptive immune system [2]. Thus, a better understanding of NHEJ could lead to improved therapies for cancer patients treated with radiation therapy and DSB-inducing chemotherapeutic agents, lead to a better understanding of the causes of radiation sensitivity and resistance and inform on mechanisms of severe combined immunodeficiency with radiation sensitivity (RS-SCID). NHEJ is also important for maintaining genome stability, which is key to cancer prevention and healthy ageing [1]. In addition, better understanding NHEJ has important implications for emerging gene editing techniques that rely on creation and repair of DSBs using CRISPR/Cas9 [3]. In this review, we will describe how recent structures of NHEJ complexes have enhanced our understanding of how NHEJ proteins detect, process and repair IR-induced DSBs. Particular emphasis will be placed on recent structures involving the DNA-dependent protein kinase catalytic subunit (DNA-PKcs), an important and druggable target in NHEJ.

## Non-homologous end joining

For mechanistic purposes, NHEJ can be considered to occur in three stages, (i) detection of the DSB, (ii) processing of IR-induced damaged or modified DNA ends to make them suitable for ligation, and (iii) ligation of the broken DNA ends. The main players in NHEJ have been known for many years, but structural insights into how these proteins interact to form large, multiprotein complexes have only recently begun to emerge [4]. In the next section, we will provide a brief overview of the main players of NHEJ before describing recent studies that reveal how they interact to repair IR-induced DSBs.

- Detection: DSB ends are detected by the Ku70/80 heterodimer which forms a basket-shaped structure with a preformed ring that encircles a molecule of double stranded (ds)DNA [5]. In addition, both Ku70 and Ku80 contain unique regions, including a C-terminal SAP domain in Ku70 [6], and the Ku80 C-terminal region (Ku80 CTR, residues 565–732) which contains a conserved motif at the extreme C-terminus (residues 720–732) shown to interact directly with DNA-PKcs [7–9].
- Processing: IR leads to multiple forms of DNA damage including DNA ends with non-ligatable groups. Thus, before DNA ends can be re-ligated, this damage must be repaired and ligatable end groups restored. DSB end processing is thought to involve polynucleotide kinase phosphatase (PNKP), which has 5′-DNA kinase and 3′-DNA phosphatase activity [10] and interacts with XRCC4 (X-ray cross-complementing protein 4) through its N-terminal FHA (fork-head associated) domain [11], Artemis/DCLRE1C/SNM1C, a nuclease that interacts with DNA-PKcs and is essential for opening DNA hairpins during V(D)J recombination [12], and DNA polymerases µ and λ, which interact with Ku through their BRCT domains [13,14]. Other proteins such as APLF (Aprataxin and PNKP-like factor), which interacts with XRCC4 through a FHA domain and Ku through a Ku binding motif (KBM), and PAXX (paralog of XRCC4 and XLF), which also interacts with Ku through a KBM, may also be involved in stabilizing NHEJ complexes on DNA [15,16]. The Werner Syndrome Helicase (WRN) which interacts with Ku can promote NHEJ through its helicase and nuclease activities and may also play a role in end processing [17,18].
- Ligation: DSB end ligation in NHEJ requires DNA-Ligase IV which exists in complex with a homodimer of XRCC4. XRCC4 is composed of a globular head domain, a coiled–coil stalk and a disordered C-terminal tail [19], and interacts with the tandem BRCT domain of DNA-ligase IV via its coiled–coil domain [20]. XRCC4 also interacts with the related protein XLF (XRCC4-like factor) though its head domain [21,22] and XRCC4 and XLF can also form extended oligomers through head-to-head interactions [21–23].

## DNA-PKcs in NHEJ

**Figure 1.**
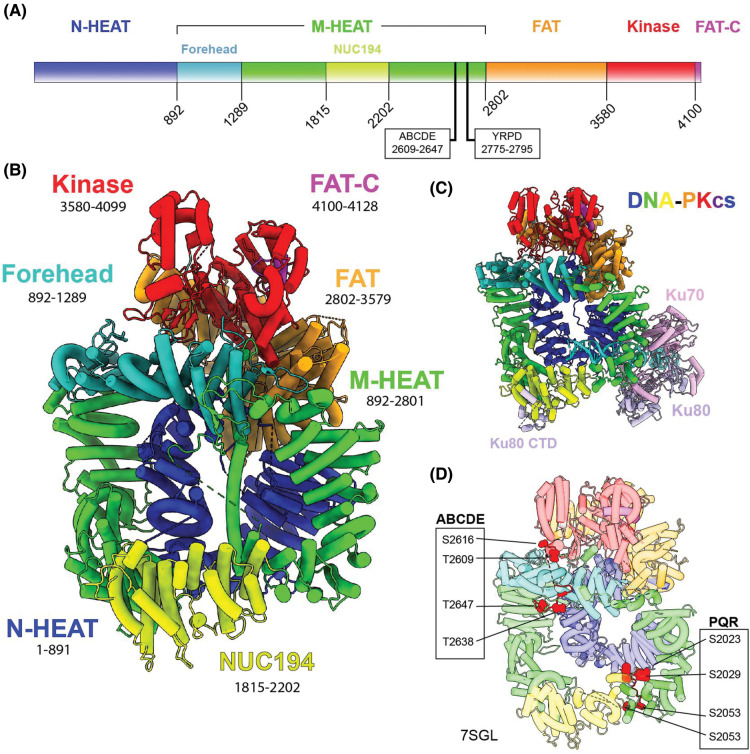
Architecture of DNA-PKcs. (**A**) Schematic of DNA-PKcs showing the location of the N-HEAT, M-HEAT, FAT, kinase and FAT-C domains as described in [26]. Also shown is the location of the ABCDE phosphorylation site cluster (residues 2609–2647), the forehead domain (residues 892–1289), the NUC194 domain (residues 1815–2202) and the YRPD motif (residues 2775–2795) [13]. (**B**) Overall structure of DNA-PKcs taken from the LR complex [54]. Domains are colored according to schematic in Figure 1A. (**C**) Structure of the DNA-PK complex including DNA-PKcs, Ku70/80, and DNA. (**D**) Location of ABCDE and PQR phosphorylation clusters in DNA-PKcs from the Artemis bound structure [33]. Residues S2612 and T2620 from the ABCDE remain unresolved. The ABCDE cluster undergoes a disordered to ordered transition after DNA-PKcs is phosphorylated, as seen in both the Artemis bound state and the LR-ATP complex (see Figure 3B).

DNA-PKcs was discovered as a protein kinase that interacts with Ku70/80 in the presence of dsDNA [24,25]. DNA-PKcs is composed of an N-terminal HEAT (Huntingtin, Elongation Factor 3, A subunit of protein phosphatase 2A, Target of Rapamycin/TOR) domain (residues 1–891), a central HEAT region, often termed the circular cradle (residues 892–2799) and a C-terminal kinase domain (residues 3580–4100) that is flanked by conserved FAT (FRAP, ATM and TOR) and FATC (FAT-C terminal) domains [26] (Figure 1A). X-ray crystallography and cryo-electron microscopy (cryo-EM) revealed that the FAT-kinase and FATC domains of DNA-PKcs form a head or crown that sits atop a large alpha-solenoid ring formed by the N-HEAT and M-HEAT domains [26,27] (Figure 1B).

DNA-PKcs is a member of the phosphatidylinositol-3 kinase-related protein kinase (PIKK) family of serine/threonine protein kinases and shares many features with the related proteins, ATM (Ataxia Telangiectasia Mutated) and ATR (ATM and Rad3-related) [28]. However, unlike ATM and ATR, DNA-PKcs is absent from several model organisms including *C. elegans*, *Arabidopsis thaliana* and the Dikarya yeast *S. cerevisiae* and *S. pombe*, which has led to the assumption that DNA-PKcs is a vertebrate-specific PIKK. However, recent bioinformatics studies have revealed that *PRKDC*, the gene encoding DNA-PKcs, is present in a wide variety of invertebrates, ciliates, molds as well as non-flowering plants, suggesting that it has a far more ancient lineage than previously thought [29,30]. Bioinformatics analysis also identified several highly conserved sequences in DNA-PKcs, including the YRPD motif, predicted to play an important role in DNA-PKcs function, as described below [29].

Early biochemical studies revealed that DNA-PKcs interacts with Ku70/80 on dsDNA to form the DNA-activated protein kinase, DNA-PK [25], an interaction mediated by residues 720–732 at the extreme end of the KU80 CTR [7]. Recruitment of DNA-PKcs to DNA bound Ku was also shown to push Ku inwards, away from the end, so that DNA-PKcs itself occupies the position at the extreme end of the DSB [31]. This conformation was confirmed by the cryo-EM structure of the DNA-PKcs, Ku70/80 complex bound to a single dsDNA end, i.e. the DNA-PK holoenzyme complex [32] (Figure 1C). Complexes of Artemis with the DNA-PK complex have also been captured [33,34]. Here, the N-terminal nuclease domain of Artemis interacts with DNA-PKcs by insertion between the N-HEAT and M-HEAT domains and between DNA-PKcs and Ku70/80 [33].

### DNA-PKcs autophosphorylation

Using purified DNA-PKcs and Ku70/80, it was shown that DNA-PKcs undergoes autophosphorylation on multiple sites which leads to its dissociation from Ku-bound DNA and loss of kinase activity [9,35–37]. The most well-studied autophosphorylation sites are the ABCDE cluster (threonines 2609, 2620, 2638 and 2647 and serines 2612 and 2624), located in a flexible loop unresolved in most existing structures due to its disorder, and the PQR cluster (serines 2023, 2029, 2041, 2053, and 2056) located in the NUC194 domain of DNA-PKcs [35,38–41] (Figure 1A,D). Disruption of the ABCDE phosphorylation sites renders cells more radiation sensitive than cells lacking DNA-PKcs [38]. Moreover, blocking phosphorylation of the ABCDE sites reduces processing of DSB ends and blocks the other major DSB repair pathway, Homologous Recombination Repair (HR) [39]. Conversely, phosphorylation of the PQR cluster makes DNA ends more accessible and enhances the use of HR, thus phosphorylation of the ABCDE and PQR clusters have opposite effects on DNA-PKcs function in NHEJ [39]. While most studies agree that phosphorylation of S2056 in the PQR cluster is DNA-PK-dependent *in vivo* (consistent with autophosphorylation) [42,43], ATM and ATR have also been reported to phosphorylate the ABCDE cluster [43–45].

## Mechanism of NHEJ

Based on *in vitro* and *in vivo* experiments from many labs over the past 30 years, a model for NHEJ emerged in which the Ku70/80 heterodimer binds to the ends of the DSB, followed by recruitment of DNA-PKcs, to tether the DSB ends together [1,4,46,47]. These models suggested that DNA-PKcs then undergoes autophosphorylation, leading to its dissociation, leaving the DSB ends available for processing, for example by PNKP, Artemis and DNA polymerases µ and λ. Once ligatable end-groups were restored, the ends would be ligated by the XRCC4-Ligase IV complex [47]. However, a critical question for this model was when were XRCC4-DNA ligase IV, XLF and the processing proteins recruited to the DSB site? Indeed, subsequent models proposed assembly of a multiprotein complex at the DSB, rather than a sequential pathway for NHEJ [48–50]. However, precisely how the various proteins interacted was unknown. Also, unknown was how the NHEJ proteins keep the DSB ends available for processing while keeping them in proximity for the final ligation step.

An answer to this puzzle came from elegant experiments by Loparo et al. [51] who, through single-molecule (sm)FRET studies in *Xenopus* cell extracts, showed that the DSB ends are initially bound by a what they termed a ‘long-range' (LR) complex in which the DSB ends are held more than 100 Å apart and a ‘short-range' (SR) complex, formation of which required the catalytic activity of DNA-PKcs as well as the presence of XLF, XRCC4 and DNA-ligase IV. In the SR complex, the DSB ends were held in close proximity, likely capable of synapsis [51]. Moreover, inhibition of DNA-PKcs prevented the formation of the SR complex suggesting that the initial LR complex transitioned into the SR complex [51]. Critically, their experiments also revealed that these NHEJ complexes contained a single homodimer of XLF [52].

### Structure of a long-range complex

**Figure 2.**
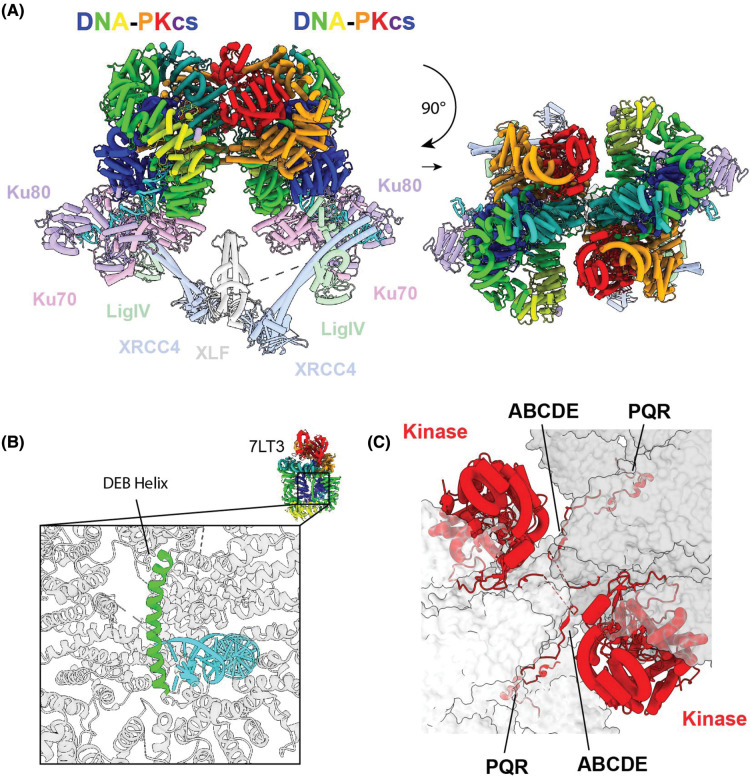
Architecture of the LR complex. (**A**) Structure of the LR complex from [51] with DNA-PKcs colored as in Figure 1A. Ku70 and 80 are in pink and lavender, XRCC4 in light blue, XLF in gray and the tandem BRCT domain of DNA-ligase IV is in light blue. The dsDNA is in turquoise. This unit is referred to as the XLF-XRCC4-DNA ligase IV scaffold in the text. The structure on the right has been rotated by 90° to show the relative arrangement of the two DNA-PKcs molecules such that the kinase domain of one (red) is in proximity to the tentative position of the disordered ABCDE loop of the opposite molecule. (**B**) Location of the DEB helix in the LR complex, which becomes ordered and occupies the position at the double-strand DNA termini. (**C**) Closeup of kinase domains of DNA-PKcs in the LR complex with the ABCDE and PQR clusters from 7SGL superimposed. Each kinase domain sits directly opposite the opposing ABCDE phosphorylation clusters.

Subsequently, cryo-EM studies by He, Blundell and colleagues put these findings into a structural context [53,54]. Both groups described NHEJ complexes containing two Ku heterodimers, each interacting with a DNA-PKcs molecule, assembled at two DSB ends (Figure 2A). Consistent with earlier biochemical experiments, DNA-PKcs interacted directly with the DSB ends, while Ku70/80 was located distal from the DSB. In the LR complex, the DSB ends interacted directly with a DNA end binding helix (residues 2736–2767) within each DNA-PKcs monomer (Figure 2B). Two DNA-PKcs monomers interacted across the two DSB ends, described as a ‘pre-synaptic complex', mediated in part by the conserved forehead domain and YRPD motif of DNA-PKcs [54,55]. In the LR complex, the YRPD motif (residues 2773–2795) interacted directly with a YRPD-interacting motif (YRPDI, residues 2569–2585) in both DNA-PKcs monomers. In neither structure, was electron density observed for the region containing the ABCDE phosphorylation sites, consistent with this region being disordered [56]. However, the kinase domain of each DNA-PKcs was positioned such that it was predicted to be able to phosphorylate the ABCDE loop of the opposite monomer (Figure 2C). Thus, in this conformation, phosphorylation of the ABCDE loop could likely occur *in trans* (inter-molecular) [53,54]. Given the extensive interaction interface between the two DNA-PKcs monomers, it is unlikely that ATM or ATR could contribute to DNA-PK phosphorylation in this conformation [55].

Significantly, both studies found that two XRCC4 homodimers, each interacting with the tandem BRCT domain of its associated DNA-ligase IV, interacted with XLF through their head domains to form a W-shaped scaffold [53,54] (Figure 2A). In this complex, the DSB ends were staggered and held 115Å apart, consistent with the LR complex from earlier smFRET and mass spectrometry studies [51–54,57]. Interestingly, a small region of the C-terminal tail of XRCC4 was observed to bind a cleft in DNA-PKcs in the LR complex [54], which was later revealed to also bind a portion of Artemis’ C-terminal tail [33], suggesting that Artemis and XRCC4 may compete for binding to DNA-PKcs, or interact in a sequential manner with the LR complex.

### Structure of a DNA-PKcs independent short-range complex

He and colleagues also described a second complex containing Ku70/80, XLF, XRCC4-DNA ligase IV assembled on dsDNA in the absence of DNA-PKcs, termed the SR complex. In this complex, the W-shape scaffold was retained but rotated such that the DNA ends were aligned for ligation. Furthermore, a single catalytic domain of DNA-ligase IV was present at the DNA break [54]. DNA-ligase IV is composed of an N-terminal catalytic domain which is flexibly tethered to XRCC4 via its tandem BRCT domain [58]. Moreover, it is a single turnover enzyme that needs to be adenylated prior to ligation [59]. Together, these findings suggest that the two catalytic domains of DNA-ligase IV bind in a sequential manner to join one strand of the DSB then the other [54].

### Effects of DNA-PKcs autophosphorylation on the structure of the LR complex

**Figure 3.**
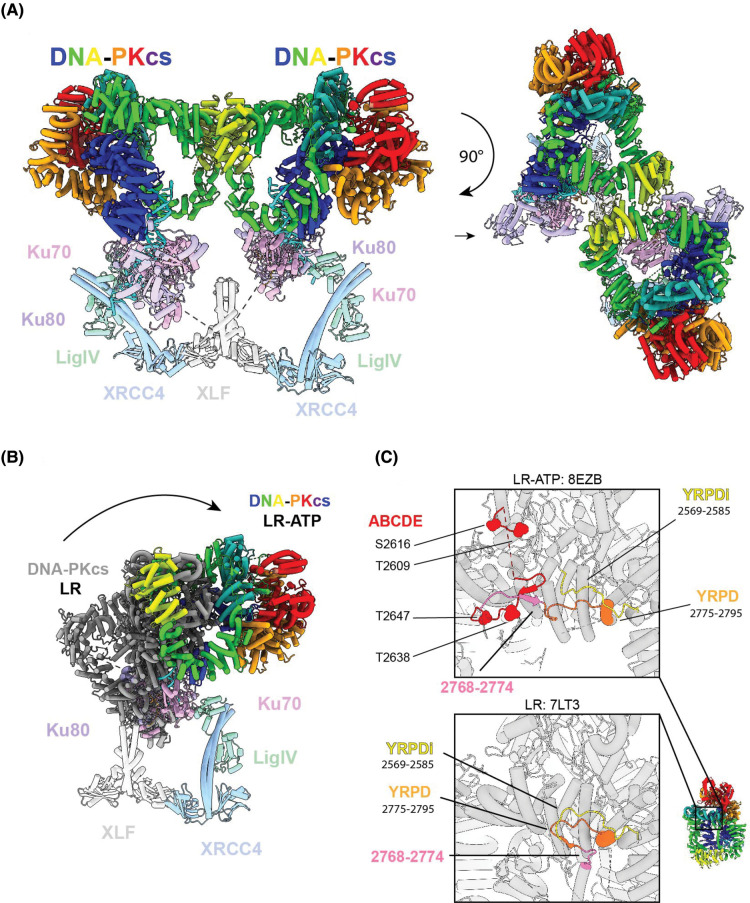
Architecture of the LR-ATP state. (**A**) Structure of the LR-ATP complex from [58], colored as in Figure 1A, showing the outward rotation of DNA-PKcs, the inward rotation of Ku70/80 and the new interaction of the two NUC194 domains. The structure on the right is rotated by 90°. (**B**) Rotation of DNA-PKcs relative to the XRCC4-XLF scaffold between LR and LR-ATP states. (**C**) Closeup of the disordered-to-ordered transition in the ABCDE cluster observed between the LR and LR-ATP states. The YRPD and YRPD-interacting motifs also undergo substantial conformational rearrangement, as shown.

He and colleagues have now generated an additional LR complex formed in the presence of DNA-PKcs, Ku70/80, dsDNA, PAXX, XLF and XRCC4-DNA-ligase IV [60]. Incubation of this complex (as well as the original LR complex) with ATP, but not the slowly hydrolyzable analog ATP-γ-S, resulted in massive reorganization of the complex [60] (Figure 3A). In this new complex, termed LR-ATP, the two DNA-PKcs molecules are rotated outwards by ∼60° (Figure 3B). Additionally, DNA-PKcs and Ku rotate inwards along the DNA double-helix by ∼90° and 30°, respectively [60]. In this new arrangement, the previously observed interactions between the two forehead domains of DNA-PKcs, the highly conserved YRPD/YRPDI motifs, the interaction of DNA-PKcs with the Ku80 CTR and the interaction of each DNA-PKcs’ DNA end binding helix with the DSB ends [54] were all disrupted. In LR-ATP the main site of interaction between the two DNA-PKcs molecules was the NUC194 domains (Figures 1A, 3A). Moreover, in LR-ATP, the DSB ends were exposed and 88 Å apart [60]. In addition, the circular cradle had undergone a dramatic opening, and the loop containing the ABCDE phosphorylation sites had undergone a disorder-to-order transition allowing it to be observed interacting with the forehead domain (Figure 3C) [60], similar to the conformation of autophosphorylated DNA-PKcs in the previously described DNA-PKcs-Ku-DNA-Artemis complex [33]. Together, these studies support a critical role for DNA-PKcs autophosphorylation in facilitating the transition from the initial LR complex through LR-ATP into the SR complex where DNA-ligase IV can rejoin the breaks.

### Additional DNA-PKcs containing complexes

**Figure 4.**
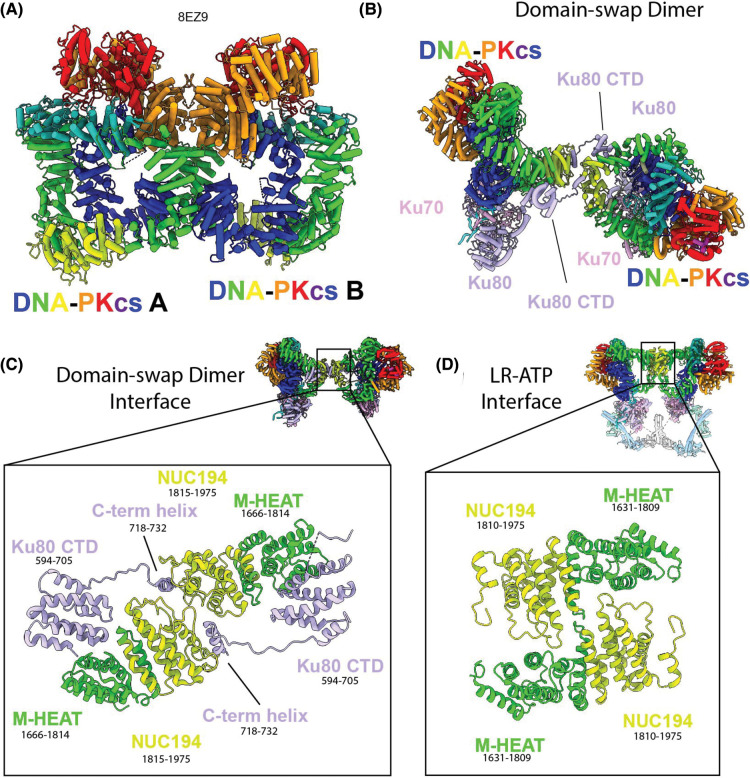
Other dimeric forms of DNA-PKcs. (**A**) Structure of the apo DNA-PKcs dimer captured in the absence of other NHEJ factors [60]. The interaction site occludes Ku70/80 binding, implying that this state is an inactive form of the kinase. (**B**) Domain-swap dimer [64]. (**C**) Closeup of DNA-PK dimer interface in domain-swap dimer. Dimeric interactions are mediated by C-terminal region of Ku80 interacting *in trans* with NUC194 and M-HEAT regions of opposing DNA-PKcs. (**D**) Comparison of the same region of interaction in the LR-ATP complex. The interaction site is shifted relative to the DNA-PK dimer, with the NUC194 of each copy of DNA-PKcs interacting with an opposing region of the M-HEAT domain.

Like the other DNA repair-related PIKKs, ATM and ATR [61–63], DNA-PKcs (in the absence of Ku70/80, XRCC4-XLF-DNA Ligase IV and DNA) has also been observed in an apo-homodimeric state [58]. The dimeric interface responsible for this new interaction includes regions of the N-HEAT, M-HEAT, and FAT domains, some of which overlap with the interface that mediates the interaction with Ku70/80, which sterically occludes the formation of the DNA-PK complex (Figure 4A) [60]. Although the function of this ostensibly inactive state is unclear, it is possible it could serve as a reservoir of DNA-PKcs in the nucleus.

DNA-PKcs and Ku (in the absence of XRCC4-XLF-DNA Ligase IV) have also been shown to form a ‘domain-swap dimer' in which the CTR of one Ku80 molecule interacts with the DNA-PKcs molecule on the opposite side of the DSB (Figure 4B,C) [64]. Intriguingly, in this complex, the DSB ends are also staggered and 115 Å apart as seen in the LR complex [51] (Figure 4B,C). Indeed, the orientations of each copy of DNA-PKcs relative to the other in the DNA-PK dimer is strikingly similar to that observed in the LR-ATP complex [60]. A comparison of the interfaces reveals that almost identical regions of DNA-PKcs are involved in forming these interactions (Figure 4B,C). However, the DNA-PK domain-swap dimer relies heavily on interactions between the Ku80 CTR and DNA-PKcs, which are completely absent from the LR-ATP state. Furthermore, the LR-ATP complex features interactions between NUC194 and M-HEAT regions of opposing DNA-PKcs molecules, while only the NUC194 regions of DNA-PKcs are juxtaposed in the DNA-PK domain-swap dimer.

Recently, elegant mutational studies by Meek and colleagues suggest that the LR complex and the domain-swap dimer support opposite end processing activities, with the LR complex promoting fill-in end processing, while the domain-swap dimer promotes DNA end resection [65]. They propose that the formation of either the domain swap or LR complex is dictated by the type of end processing required at the DSB; the domain-swap dimer forming in cases where end resection is required, and the LR complex assembling when NHEJ polymerases are needed [65]. Given the similarities between the DNA-PK dimer and LR-ATP state, specifically the orientation of DNA-PKcs in each complex, the observations by Meek and colleagues may also yield insight into the function of the LR-ATP state. As described above, DNA-PKcs in the LR-ATP adopts a similar ‘open’ conformation as observed by Yang et al. [33] in their DNA-PK-Artemis structure, which accommodates Artemis’ nuclease domain. These structural features, coupled with the observation that the DNA-PK dimer preferentially supports end resection raises the possibility that Artemis may be able to function in the LR-ATP state as well.

Together, these new structural studies suggest that DNA-PKcs can participate in multiple complexes, adopting many different orientations and conformations. Moreover, higher-order structures composed of multiple DNA-PK-containing complexes have also been observed [66] suggesting that NHEJ complexes might coat extended regions of DNA in a manner similar to Rad51 filaments in HR [67]. The different structures may present different surfaces for phosphorylation and/or interaction with binding partners. Therefore, different modes of DNA-PKcs phosphorylation may occur depending on which complex it is associated with. Similarly, different complexes may facilitate different modes of end processing, as proposed by Meek et al. [65].

## Summary and outstanding questions

In summary, the past few years have yielded a plethora of exciting and informative NHEJ structures that provide the first glimpses into how NHEJ proteins assemble at a DSB, tether the DSB ends and subsequently make them accessible for ligation. Nevertheless, many questions remain. For example, when and how are the processing enzymes recruited to the NHEJ complex? For example, the FHA domains of PNKP and APLF interact with XRCC4 [11] while APLF and DNA polymerases µ and λ interact with Ku [68,69]. One possibility is that such proteins are recruited to the W-shaped scaffold via their interacting domains, while their catalytic domains remain flexibly attached, allowing access to the DSB ends at a distance as seen for DNA-ligase IV [54,55]. Another critical question is when does end processing takes place? Are DSB ends processed after DNA-PKcs autophosphorylation when DNA-PKcs is in the LR-ATP conformation (Figure 3A), or does DNA-PKcs need to completely dissociate from the complex, as in the SR complex before ends are processed? [54]. Similarly, how are processing and ligation synchronized? Does the catalytic domain of DNA-ligase IV sample the unprocessed DSB ends until it can form a stable complex with the processed ligatable ends? Also, when is Artemis recruited to the NHEJ complex? Does the DNA-PK-Artemis complex exist with just DNA-PKcs and Ku as shown in [33], or can it be recruited as part of the larger LR complex? Interestingly, Artemis also interacts with DNA-ligase IV [70] which suggests interaction with the LR complex or handover from a DNA-PK-Artemis or LR-Artemis complex to the SR complex. Other important questions include how NHEJ complexes interact with other components of the DNA damage response and whether DNA-PKcs is dephosphorylated after release from LR-ATP and if so, by which phosphatases. DNA-PKcs has been shown to interact with protein phosphatases PP5 [71] and PP6 [72], but whether these phosphatases are required for dephosphorylating DNA-PKcs at DSB sites is not known.

Finally, as a protein kinase required for repair of IR-induced DSB through NHEJ, DNA-PKcs is an attractive target for therapeutic intervention using small molecule inhibitors [73]. Moreover, DNA-PKcs mRNA is overexpressed in many tumor types [29], and DNA-PK inhibitors are in clinical trials for a variety of solid tumors [74]. Intriguingly, increasing evidence suggests that DNA-PKcs is required for additional cellular processes as diverse as metastasis [75], ribosomal RNA processing [76], metabolism [77], ageing [78,79] and mitochondrial function [80]. How DNA-PKcs functions in these situations is not well understood and could require the formation of additional multi-component complexes, as shown recently for DNA-PKcs and mitochondrial proteins [80]. Clearly, a better understanding of DNA-PKcs function along with detailed information on the structures of various DNA-PKcs-NHEJ and non-NHEJ complexes could lead to the development of more specific inhibitors of NHEJ as well as DNA-PK inhibitors with a wide variety of clinical applications.

## Perspectives

- Non-Homologous end joining (NHEJ) is the major pathway for the repair of ionizing radiation-induced DNA double-strand breaks (DSBs) in human cells. A better understanding of NHEJ has major implications for improving treatment of cancer patients with radiation therapy and chemotherapy as well as better understanding other repair and non-repair processes.
- Although individual components have been identified and well-studied, how NHEJ proteins combine and interact at sites of damage to repair DSBs has been unclear. Here, we discuss recent structural advances that show how NHEJ proteins assemble at DSBs, and reveal how DNA-dependent protein kinase catalytic subunit (DNA-PKcs) autophosphorylation regulates the transition from protected to ligatable DNA ends.
- These new studies reveal how DSB ends are detected, protected and ligated during NHEJ. How processing enzymes that repair ionizing radiation (IR)-damaged DNA ends are recruited to and function at these newly described NHEJ machines remains to be determined.

## Abbreviations

ATM: ataxia telangiectasia mutated
DNA-PKcs: DNA-dependent protein kinase catalytic subunit
DSBs: double-strand breaks
FHA: fork-head associated
IR: ionizing radiation
KBM: Ku binding motif
LR: long-range
NHEJ: non-homologous end joining
PIKK: phosphatidylinositol-3 kinase-related protein kinase
PNKP: polynucleotide kinase phosphatase
SR: short-range
TOR: target of rapamycin
